# The role of directional interactions in the designability of generalized heteropolymers

**DOI:** 10.1038/s41598-017-04720-7

**Published:** 2017-07-10

**Authors:** Chiara Cardelli, Valentino Bianco, Lorenzo Rovigatti, Francesca Nerattini, Luca Tubiana, Christoph Dellago, Ivan Coluzza

**Affiliations:** 1 Faculty of Physics, University of Vienna, Boltzmanngasse 5, A-1090 Vienna, Austria; 2 Rudolf Peierls Centre for Theoretical Physics, University of Oxford, 1 Keble Road, Oxford, UK

## Abstract

Heteropolymers are important examples of self-assembling systems. However, in the design of artificial heteropolymers the control over the single chain self-assembling properties does not reach that of the natural bio-polymers, and in particular proteins. Here, we introduce a sufficiency criterion to identify polymers that can be designed to adopt a predetermined structure and show that it is fulfilled by polymers made of monomers interacting through directional (anisotropic) interactions. The criterion is based on the appearance of a particular peak in the radial distribution function, that we show being a universal feature of all designable heteropolymers, as it is present also in natural proteins. Our criterion can be used to engineer new self-assembling modular polymers that will open new avenues for applications in materials science.

## Introduction

Gaining control of self-assembly processes is key for the generation of smart materials, with applications ranging from energetics^1^, to photonic crystals^2^ and biomimetic scaffolding^3^. Among the most promising and versatile classes of systems that undergo self-assembly are polymers composed of monomers of different species, also known as heteropolymers^4–7^. Remarkable examples are natural self-assembling systems such as DNA^8^, RNA^9^ and, in particular, proteins^10,11^. Their extremely rich self-assembling behaviour is mainly controlled by the *sequence* of chemically different building blocks, or monomers, that compose the polymer. The collection of all possible monomer types is the *alphabet* of the system. For proteins, different sequences of the same alphabet of 20 amino acids lead to the huge number of proteins expressed in nature, making the alphabet extremely versatile. Indeed, it is the specific sequence that drives a heteropolymer to uniquely collapse (fold) into a target conformation, providing precise control over the resulting structure.

Artificial heteropolymers synthesis and manipulation is at an advanced stage, as demonstrated by the numerous materials based on block copolymers^4^. Typically, block copolymers are synthesised from two monomer types (AB copolymers), but there are examples of linear chains synthesis with an alphabet size of 4 (ABCD block copolymers)^6,7^ or star polymers with up to 7 types^12^. The different types are organized in patterns of blocks of the same repeated monomer, from two up to five blocks^4^, and by controlling the relative length of the blocks it is possible to realize rich phase diagrams^4,5,13–19^).

However, it is extremely difficult to drive the folding of a single chain towards a very specific structure, especially with the large variety of different structures and the high accuracy, fractions of inter-monomer distances, obtained by biopolymers^11,20–23^ that is necessary for many applications (*e*.*g*. catalysts). Recently, efforts to overcome this limitation have been made^24–28^, in particular by Khokhlov *et al*.^29^, where copolymer chains have been iteratively “painted” to undergo a hydrophobic-hydrophilic micro-phase separation and by Moreno *et al*.^30^, who have engineered specific hydrophilic-hydrophilic patterns to produce a similar micro-phase separation. However, contrary to biopolymers, these two methodologies do not provide control over the detailed shape of the target structure.

According to mean field theories (MFT)^31–33^ it should be possible to construct artificial heteropolymers that, similarly to proteins, drive the collapse of specific sequences into given target structures. The identification of such sequences is normally referred to as *design* and, in what follows, we define *designability* as the property of a heteropolymer to have at least one heterogeneous sequence that reliably folds into a given stable target structure^33^. A clear review of the MFT mentioned above can be found in the seminal works of Pande *et al*.^33^, who showed that the designability of a heteropolymer increases with the alphabet size and decreases with the conformational entropy per bead. Thus, the designability of a heteropolymer can be enhanced following two strategies: i) by increasing the alphabet size as in Go-models^34^, where each amino acid can specifically interact only with a subset of residues and, ii) by decreasing the configurational entropy per particle, i.e. reducing the number of possible configurations.

A straightforward way to adopt the second strategy is represented by designing lattice heteropolymers^32,35–39^, in which the number of possible configurations (thus the configurational entropy) is reduced by the topology of the lattice.

In order to adopt the second strategy for off-lattice polymers a possibly effective way is to introduce directional interactions, *i*.*e*. interactions that depend on the mutual orientations of the monomers, in addition to the heterogeneous isotropic interactions. In this way, the conformational entropy per bead decreases because of constraints on the mutual orientations. In fact, the directionality of the interactions introduces a frustration in the system that “pre-sculpts” the configurational space of the system, which will contain just a fraction of the total compact configurations. Another effective way to reduce the conformational entropy is to increase the chain stiffness. An important contribution in this direction is represented by the “tube protein model”, developed by Maritan and co-workers^40–44^, in which control of the conformational free-energy landscape is achieved by tuning the total hydrophobicity and the chain stiffness of short polymers, which can be made designable in some specific cases^41,43^. Natural proteins take advantage of both routes by exploiting the directionality of the hydrogen bonds and the geometry of the backbone^42–45^. From an experimental standpoint, in fact, decreasing the conformational entropy via these two routes is more feasible then increasing the alphabet like in Go-Models, which might be the reason why directional interactions (such as hydrogen bonds) are so important in biology.

**Figure 1:**
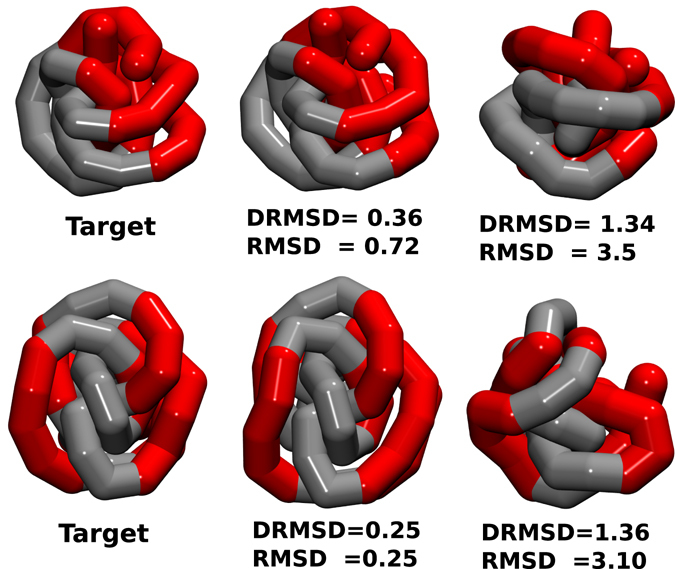
Functionalized patchy polymer structures. Simulation snapshots showing the target structures with the desired functionalizations (left), the final folded structures obtained using our design procedure (centre) and examples of misfolded structures (right), the last two aligned with respect to the correspondent target structure. The values of *DRMSD* (distance root mean square displacement, see Eq. 3) and *RMSD* (root mean square displacement see Eq. 4) with respect to the target structure are in units of the bead radius, that for proteins has been seen to correspond to approximately 2 Å, so in protein units the two folded structures would have a *RMSD* with respect to the target structure in the range 0.5–1.4 Å and the two molten globule structures in the range 6–7 Å. Such functionalized polymers can, in turn, be used as Janus (one-patch) particles (top) or triblock Janus (two patches) particles (bottom).

In this work, we show that the presence of directional interactions in a generalised heteropolymer allows for the realisation of compact structures with functionalized regions on their surfaces with an accuracy comparable to natural catalysts, *i*.*e*. of the order of a fraction of amino acids distances. To give an idea of the huge potential of such an approach, in Fig. 1 we present simulation snapshots of heteropolymers that self-assemble with high-accuracy into predetermined structures: two spherical objects with patterned (functionalized) surfaces. The sequences we design are able to make the two heteropolymers reliably fold into a Janus-like (top) and a triblock-Janus-like (bottom) object with *DRMSD* (distance root mean square displacement see Eq. 3) of 0.36 and 0.25 with respect to the target structure which correspond to an *RMSD* (root mean square displacement see Eq. 4) of 0.72 and 0.25 with respect to the target structure, respectively. Depending on the chemical nature of the functionalization, such folded structures could exhibit catalytic functions or be used as building blocks in a hierarchical self-assembling process^46^. Of course, these applications would also require a precise control of inter-chain interactions in order to avoid amorphous aggregations^47^.

However, in order to exploit the folding of artificial heteropolymers for applications, we ought to first address the following question: “Can we predict *a priori* whether an engineered heteropolymer is designable?” In other words, if one engineers heteropolymer architectures that differ in terms of number, geometry or nature of the directional interaction, is it possible to predict a priori which ones are designable (i.e., for which ones one can find at least one specific sequence that drives the system to fold into a given target structure)?

**Figure 2:**
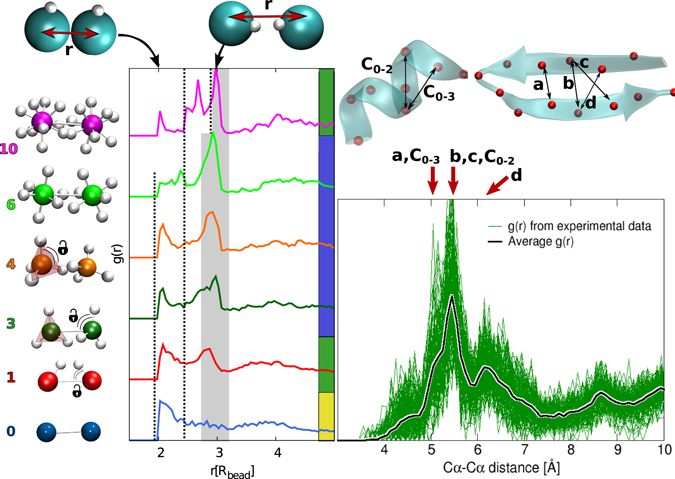
(**a**) Radial distribution functions *g*(*r*) for patchy polymers. We show one *g*(*r*) curve for each patch arrangements because we observed that it does not change significantly for different alphabet sizes. The *g*(*r*) has been averaged on the 40 most probable conformations for different patches arrangements (y-axis): white spheres indicate the patches located at the surface of the monomer. The *g*(*r*) are y-shifted for the sake of visualisation. The random packing peak is delimited by two dashed lines, while the grey band highlights the range of the directional peak. Note that for 10 patches the random packing peak splits and moves towards larger distances (Supp. Mat. Fig. 4). The ratios between the random packing peak and the directional peak areas are: 0.75 for 1 patch, 0.23 for 3 patches, 0.27 for 4 patches, 0.22 for 6 patches, and 1.33 for 10 patches. (**b**) *g*(*r*) calculated over the *C*_*α*_–*C*_*α*_ distances for 145 high resolution protein structures from the Protein Data Bank (green lines). The thick black line is the average. The first peaks dominating the *g*(*r*) correspond to the characteristic distances of the backbone hydrogen bonds, which determine the secondary structure^64^ (*α*-helix and *β*-sheet shown on top). All *g*(*r*) have been calculated by neglecting the contribution of the first neighbour along the chain.

**Figure 3:**
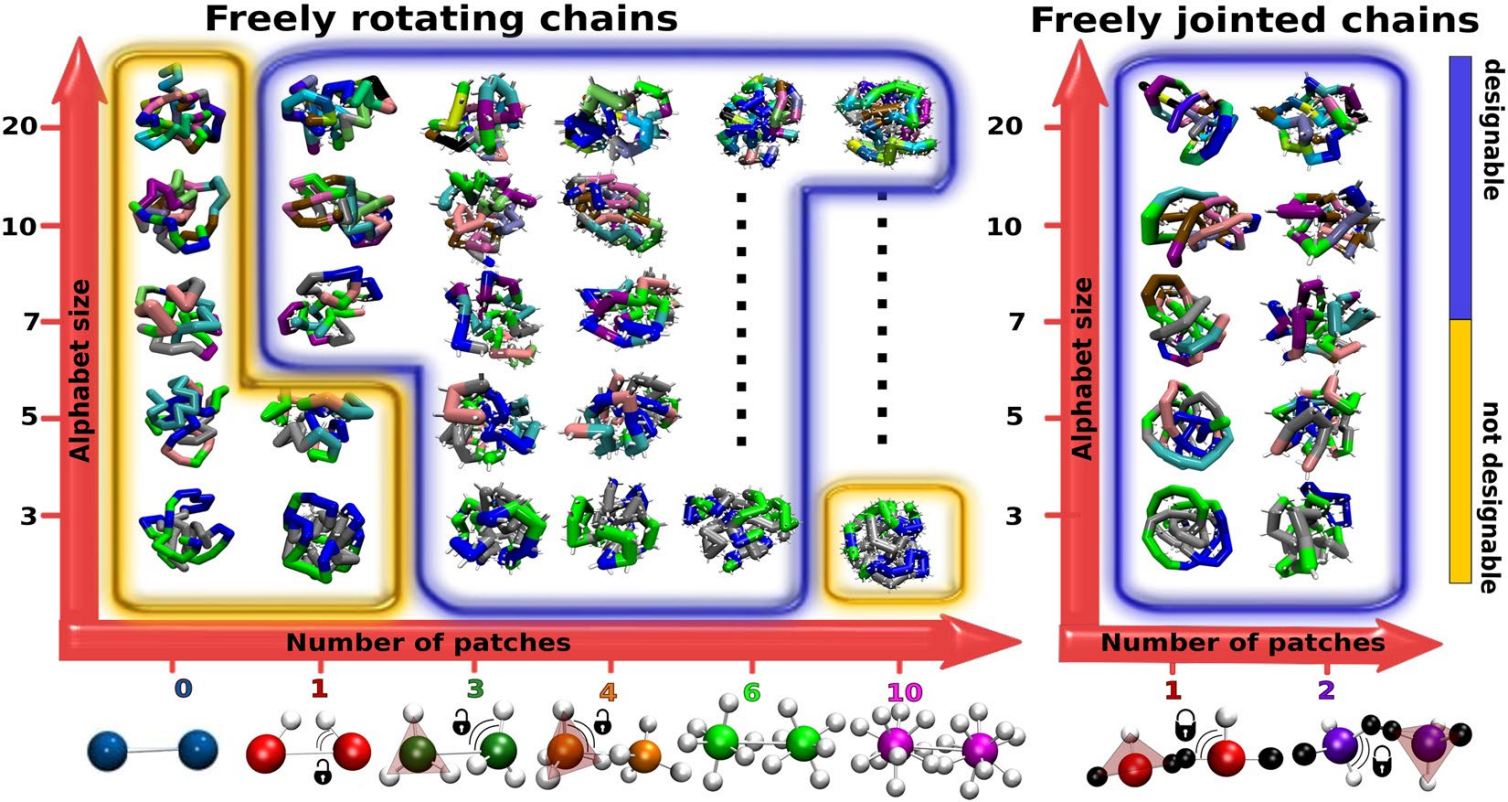
Designability diagram for different patch numbers and alphabet sizes. Within the yellow regions, the heteropolymer is not designable, while the blue regions correspond to designable systems. For each case, the chosen target structure is shown. Bottom panel: a schematic illustration of patchy polymers. White spheres indicate the patches located at the surface of the central monomer. For FRC (left) we consider from 0 (simple heteropolymer case) to 10 patches and 1 and 2 patches for FJC (right). With the exception of the one patch geometry, in all the other cases the patches are fixed on the vertices of a platonic solid or in the most symmetric way (Supp. Mat.). The two patch FJC are placed on a tetrahedron completed by the anchoring points (in black). For both models, we consider alphabet sizes *q* ranging form *q* = 3 to *q* = 20 (see Methods 1 for the definition of the interactions).

Here we show that the answer to this question is represented by a distinct peak in the radial distribution function dominating over the random packing of the heteropolymers. A minimal set of directional interactions is an effective way to induce such a peak (Fig. 2), which guarantees the designability (Fig. 3).

## Results and Discussion

Our working hypothesis is that the addition of directional interactions will result in designable chains as previously suggested by results obtained for the “patchy polymer” model^48,49^ and the “caterpillar” protein model^23,50^. In the following we will use two heteropolymer models with different chain stiffness (see Methods): the freely-rotating chain (FRC), marked by open locks in the figures, and the freely-jointed chain (FJC), marked by closed locks in the figures. Briefly, the “chemical character” of each monomer is given by a isotropic interaction potential, represented as a simple square-well like shape, with a different pre-factor (more or less attractive or repulsive) for every different pair of monomer types. The directional interaction between the patches is homogeneous along the chain and is the potential derived by Irbäck *et al*.^51^, commonly used to model hydrogen bonds (see Methods). By changing the number of patches per monomer we will probe the emergence of designability starting from a standard heteropolymer (no patches) up to chains of monomers with 10 patches. The number of patches unambiguously identifies their arrangement on their surface, which is chosen so as to yield the most symmetrical geometry (on the poles for two patches, equispaced on the equator for three patches, on a tetrahedron for four patches, *etc*). The bottom of Fig. 3 shows all the patch arrangements employed.

Strictly speaking, proving that a heteropolymer with a certain number of patches is not designable would require testing all possible sequences for the presence of a unique collapsed equilibrium structure. Here we rely on the statistical definition of designability, where the methodology of the Monte Carlo simulations SEEK, DESIGN and FOLD (SDF) was proven to be able to discriminate between designable and not-designable structures^49^. Briefly, the SDF method: (i) seeks the most designable target structure through an extensive sampling of the heteropolymer conformations and sequences (Supp. Mat. Fig. 1); (ii) designs the sequence that should optimally fold into the target structure; (iii) tests whether the designed sequence correctly folds into the target structure. If the folding fails with the most favourable target structure, the heteropolymer with a certain number of patches is labelled as non-designable. More details can be found in Sec. Methods 1. Here we apply the SDF method to chains made of 50 monomers. From now on, all the distances will be in units of the bead radius *R*_*bead*_ and the energies in units of *k*_*B*_*T*_*Ref*_, where *T*_*Ref*_ is a reference temperature that sets the scale of interactions.

**Figure 4:**
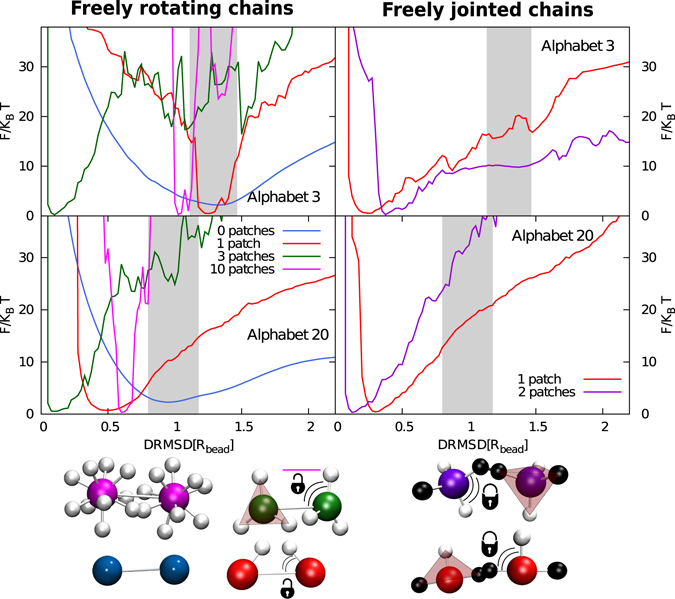
FOLDING free energy landscapes for some significant cases. The free energy is plotted as a function of the distance root mean square displacement (*DRMSD*) for FRC (left panels) and FJC (right panels), for different patch numbers and alphabet sizes *q* at temperature 0.4. *DRMSD* = 0 corresponds to the target structure. We define as molten globule region for each alphabet size (grey area) the *DRMSD* range spanning 1*k*_*B*_*T* from the position of the global minimum of the free energy of the corresponding bare polymer without patches. All global minima with *DRMSD* values below the grey area are considered correctly folded (see Methods for details), see Fig. 1 for examples of folded and molten globule structures. The geometry of the patches is reported on the bottom of the figure, with colours corresponding to the free energy curves. The global minimum of the system with 10 patches and *q* = 3 is at the border with the grey area and it has been categorised as not designable.

In Fig. 3 we show the results for the designability for all cases studied. The systems without directional interactions (patches) are not designable for any alphabet. By adding directional interactions, the heteropolymer becomes designable for a wide range of patch numbers and alphabet sizes, both for the FRC and FJC models. The emergence of designability coincides with the appearance of a peak in the pair radial distribution function—highlighted with the grey band in Fig. 2a)—located at the distance $r\simeq 3$, at which the patch-patch interaction is most favourable. The presence of such an isolated intermediate peak between the first ($r\simeq 2$) and second random close packing neighbours ($r\simeq 4$) indicates that the directional interactions are inducing a geometrical frustration in the system. The frustration strongly biases towards a subset of compact conformations. In fact, in the non-designable case without patches, the directional interaction peak is not present. For one patch, the directional interaction peak is present but is lower than the peak corresponding to the close packing. These chains with one patch are not always designable (borderline), and their designability depends on the alphabet size *q* (Fig. 3). Increasing the number of patches the directional interaction peak becomes dominating over the random packing peak, making the chains designable in a broad interval of *q* (3 to 20). Increasing further the number of patches up to 10, the random packing peak increases again, splits and moves towards higher distances (Supp. Mat. Fig. 4) due to the self-avoiding of the many patches and also the system loses directionality: this corresponds in turn to a loss of designability (Fig. 3). It is important to stress that the strength of the directional interaction is such that even the chains with one patch are always maximally bonded, implying that arbitrarily increasing the relative strength of the directional interactions will not suppress the first peak of the *g*(*r*) (Supp. Mat. Fig. 1). Thus, when the patch-patch peak dominates over the random close packing peak, the system becomes robustly designable in a broad interval of alphabet sizes *q*, both for the FRC and FJC models.

According to the MFT^33^, given an alphabet of size *q*, a system is designable when the sequence entropy per particle ln(*q*) exceeds the conformational entropy per particle *ω*. Hence, the designable–to–not-designable transition, where *ω* ~ ln(*q*), allows us to estimate *ω*. Following the diagram in Fig. 3, we estimate *ω* to be >ln(20) for 0 patches, ~ln(5) for one patch in the freely rotating chain, and <ln(3) for up to 6 patches. At 10 patches *ω* increases again to a value between ln(3) and ln(20). Since *e*^*ω*^ is related to the number of compact conformations, the latter is reduced by the patches by approximately an order of magnitude. A more precise evaluation of *ω* requires the study of intermediate values of ln(*q*) which cannot be achieved simply changing the alphabet.

The presence of the directional interaction peak is a fundamental fingerprint of designability. Indeed, we find it to be a general feature also of natural proteins. In Fig. 2b we show the radial distribution function for some characteristic examples out of 145 analysed high resolution protein structures from the Protein Data Bank. Here, the conformational space is shaped by the directionality of the hydrogen bonds, which forces the carbon C_*α*_ of the amino acids to be at the typical distances for the different types of secondary structure. Hence, the peaks analysed in Fig. 2b are equivalent to the directional interaction peak highlighted in grey in the patchy polymers. Since proteins are precisely designable because of the peculiar hydrogen-bond geometry of the backbone, we expect the latter to be an ideal template for general heteropolymers and patchy-polymers.

These results are in agreement with MFT^31–33^, which predicts that the decrease of conformational entropy allows for better sequence design. Here, we introduce the presence of a peak in the *g*(*R*) not related to the random packing as an estimate of the reduction of the conformational entropy, in order to have a simple tool to anticipate the designability of different polymer architectures. Thus, we propose the presence of such a peak as a general criterion to engineer designable polymers able to fold into unique target structures, with the same accuracy and versatility of natural proteins. The geometry of the protein skeleton is a particular choice, and our results suggest that, by following our criterion, several others can be found.

Another important result is the characterization of the transition from a not-designable to a designable number of patches (Fig. 3). We start by noting that the freely rotating chain is sensitive to the choice of the alphabet: a minimum alphabet size *q* = 7 is required to guarantee designability for any number of patches (Fig. 3). To characterise the designable–to–not-designable transition, we have performed the SDF trial for each point in the designability diagram. The last step of the SDF is the calculation of the free energy difference between structures, grouped together according to the distance root mean square displacement (*DRMSD*) from the target conformation at *DRMSD* = 0 (see Methods). The accuracy of the refolding varies considerably for each scenario and depends on the alphabet size, the number of patches and the local environment of the monomers. In Fig. 4 we show the FOLDING free energy profiles for some significant points in the designability diagram for *q* = 3 and *q* = 20 (Supp. Mat. Fig. 3). At low temperature (*T* = 0.4) all curves show a global free energy minimum located at different *DRMSD*, however, they are considered folded only if the global minimum corresponds to the folded structure and not to a disordered molten globule structure. The different nature of the global minimum can be discriminated by increasing *T* and pushing the system to unfold (Supp. Mat. Fig. 2). What is striking in the figure is the high refolding accuracy of the FJC model, with smooth folding profiles and global minima very close to the target structure. We ascribe such a high accuracy to the stronger constraint experienced by monomers in the FJC compared to the FRC model, in agreement with MFT results^31–33^. In fact, the patches in the FJC have less rotational freedom, thus the conformational entropy *ω* is decreased further. Finally, we note that such high accuracy is particularly interesting for single-patch chains, which, according to the designability criterion based on the *g*(*r*), is a borderline case (Fig. 2a).

In conclusion, we extend the concept of *designability* to heteropolymers, bridging the gap between naturally foldable biopolymers and artificial polymers. We demonstrate that there is a minimal set of ingredients that makes it possible to exert a precise control over the conformation of the folded structure. Indeed, we show that starting from a traditional heteropolymer model, we can attain designability by introducing a few directional interactions (patches). The patches make the system designable even with alphabet sizes comparable to the case of block-copolymers (3 and 5), which are synthesizable and manipulable with the current advanced technology^4,6,7^. From an experimental standpoint, in fact, small alphabet sizes are more feasible. We demonstrate that the directional interaction peak in the *g*(*R*) is a universal fingerprint for designability and can be used as a criterion to engineer new heteropolymers. This criterion will allow artificial heteropolymer to self-assemble into an enormous variety of highly exotic structures with high control over the detailed shape. This will expand the possibilities of current copolymer technology to the full potential of biopolymers, providing a way of going beyond the current protein- and DNA-based materials^22,52,53^.

Heteropolymer design could find several applications depending on the scale of the building units. At the micrometre scale, chains could be realised using colloidal particles with applications in the design of materials with new mechanical, electronic or photonic properties tailored in 3D. Colloidal particles are widely available, their interactions can be tuned to be either attractive or repulsive and can range from a few nm to several micrometres^54^. Flexible strings of polymeric colloids were made by inducing electric dipoles to line the particle up before thermal fusion^55^. Such strings were observed to collapse into a random compact configuration under influence of an induced depletion attraction. Control over the sequence can theoretically be achieved in a similar fashion as for protein synthesis where the chains are grown from a solid surface^56^. Each monomer is loaded in the sample alternating washing cycles to remove the previously unbound monomers. The limiting factor of this technique is in the bonding probability that must be extremely high in order for the growth to continue until chain lengths of 20–50 residues. Currently, we are working on strategies to overcome such limitations in close collaboration with experimental groups. At the atomic and nanometre scales, control of the sequence is in reach of current polymer synthesis technology^6,7,12^. As we show, artificial chains could be designed to place chemical groups with a precision similar to what protein can achieve^57,58^, but also to work in different environments than the ones suitable for proteins or DNA.

## Methods

**Figure 5:**
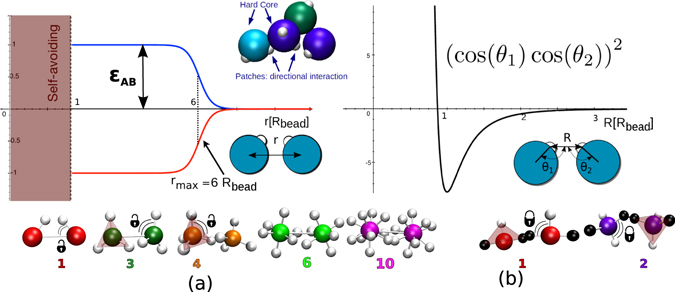
Interaction potentials. (**a**) Isotropic square-well like interaction as a function of the distance of the centers of the spherical monomers *r* (see left inset). The well depth is controlled by a pre-factor ${\epsilon }_{AB}$, which is different for each different pair of monomers. The pre-factors are grouped in an interaction matrix, the size of which depends on the number of monomer types in the range 3 to 20 (find the matrices used in the Supplementary Informations). For an alphabet of size *N*, the set of *N*(*N* + 1)/2 heterogeneous interactions are Gaussian random numbers with zero average and standard deviation of 0.33*k*_*B*_*T*_Ref_, similarly to protein coarse-grained protein models^23,65,66^, where *T*_Ref_ is a reference temperature that sets the scale of interactions. Inset: schematic illustration of patchy polymers. Small white spheres indicate the patches. (**b**) Directional potential. The radial Lennard-Jones contribution in the plot is multiplied by the directional contribution (cos *θ*_1_ cos *θ*_2_)^2^, where *θ*_1_ and *θ*_2_ are the angles between the patch vector and the distance vector (right inset).

The patchy polymer model we employ has already been proven to be able to refold artificial sequences into unique target structures^48,49^. Here we consider two standard heteropolymer models where heterogeneous isotropically-interacting monomers are bonded along the chain *via* a harmonic potential with two different anchoring geometries: (i) in the freely rotating chain (FRC) model the spring bonds the centers of the monomers; (ii) in the freely jointed chains (FJC) model the spring connects two anchoring points on the monomer surface and opposite to each other (see bottom of Fig. 3 for a sketch). An experimental realisation of the FJC model could be represented by covalently bonded chemical units or surface grafted colloidal particles. While possible experimental examples of patchy particles that could serve as monomers for the FRC model, i.e. where the patches can rotate with respect to the bead, are the lock and key colloids as in ref.^59^, the solid colloids with surface-mobile DNA linkers in ref.^60^, DNA coated emulsion droplets with mobile DNA patches^61^, colloidal particles with an induced electric dipole^55^. The isotropic and bonded interactions are complemented by non-specific additional attractions provided by patches arranged on the monomer surface. The resulting directional interaction is anisotropic in nature as it depends not only on the distance between two monomers but also on their relative orientations. The key parameters of the models are the alphabet size of the isotropic interactions, the number of patches (which is the same for all the monomers in a chain) and their the geometrical arrangement. The isotropic interaction energy *E*_*AB*_(*r*) between two different sub-units of types A and B is represented as a simple-square-well like shape (Fig. 5)

1 $${E}_{AB}\,(r)=\{\begin{array}{cc}{\epsilon }_{AB}\,[1-\frac{1}{1.0+{{\rm{e}}}^{2.5({r}_{max}-r)}}] & {\rm{i}}{\rm{f}}\,r > {R}_{bead}\\ {\rm{\infty }},= & {\rm{i}}{\rm{f}}\,r < {R}_{bead}\end{array}$$

where *r* is the distance between the centres of the beads and *R*_*bead*_ is the hard core radius, which is the same for each bead. ${\epsilon }_{AB}$ is a different pre-factor for every different pair of monomers. The cut-off distance ${r}_{max}=6{R}_{bead}$ is the distance at which ${E}_{AB}={\epsilon }_{AB}/2$ and was derived with a trial and error approach on coarse-grained proteins in the caterpillar protein model^23,50^.

As directional interaction between the patches we employ the potential derived by Irbäck *et al*.^51^, commonly used to model hydrogen bonds. It is represented by a 10–12 Lennard-Jones type potential multiplied by a factor containing the angles between the patches and the bead radius (Fig. 5), so that the energy is minimum if the patches face each other (when they are opposite to each other the radial part of the potential is ~0)

2 $${E}_{p}=s\,{\epsilon }_{p}{(\cos {\theta }_{1}\cos {\theta }_{2})}^{\nu }\,[5{(\frac{\sigma }{R})}^{12}-6{(\frac{\sigma }{R})}^{10}].$$

Here *R* is the distance of the patches as in Fig. 5 (right inset), ${\epsilon }_{p}=3.1\,{k}_{B}T$ and *ν* = 2^51^ while we set *σ* = *R*_*bead*_. The scaling factor *s* is chosen to not over favour the patch contribution over the isotropic one. If its value is too large all sequences form regular structures that depend solely on the symmetries of the patch arrangements on the beads. On the other hand, if it is too small all sequences fail to self-assemble and collapse into random glassy three-dimensional structures. Using a trial and error approach, we found 4 to be good number^49^. The neighbour beads along the chain are bonded via a harmonic spring potential.

In order to find whether the polymer is designable or not, we identify for each different number of patches and alphabet size at least one pattern (sequence) that has a global free energy minimum into a given structure. To increase the chances to find such a pattern, we first perform a SEEK MC simulation, in order to find potentially designable target structures that are not known from nature, unlike for proteins. In the SEEK, we explore at the same time different structures and sequences, and we extract a target structure from the global minimum of the free energy landscape obtained (see Supp. Mat. Fig. 1). The global minimum corresponds to the structure with the highest number of sequences that fold into it, thus the most designable one^62^. Other structures further from the global minimum might be good candidates as well, so the solution is not necessarily unique. On the contrary, if this structure will be not designable, it is highly unlikely and thus unfeasible for practical purposes to find another structure that will be designable.

The target structure is then redesigned in the DESIGN, where we explore only the different sequences maintaining the structure frozen. Here we choose the optimised sequence in the global minimum of the free energy, which in our method corresponds to a low potential energy and a high heterogeneity of the sequence.

Starting from a fully stretched structure we then perform a FOLDING Monte Carlo simulation, in order to study the self-assembling properties of this pattern. Here we explore the conformational space keeping the pattern fixed in the designed sequence.

We project the FOLDING free energy onto an order parameter, namely the root mean square displacement of the inter-particle distance (*DRMSD*) between the target structure and each sampled structure:

3 $$DRMSD=\frac{1}{N}\sqrt{\sum _{ij}\,{(|{\rm{\Delta }}{\overrightarrow{r}}_{ij}|-|{\rm{\Delta }}{\overrightarrow{r}}_{ij}^{T}|)}^{2}}$$

where ${\rm{\Delta }}{\overrightarrow{r}}_{ij}$ is the distance between the sphere *i* and *j* while ${\rm{\Delta }}{{\overrightarrow{r}}_{ij}}^{T}$ is the same distance calculated over the target structure, and *N* is the chain length (50 in our case). Most studies dedicated to proteins adopt another order parameter, the *RMSD*. This differ from the *DRMSD* in that its definition contains the positions of the atoms instead of their distances:

4 $$RMSD=\frac{1}{N}\sqrt{\sum _{ij}\,{(|{\overrightarrow{r}}_{ij}|-|{{\overrightarrow{r}}_{ij}}^{T}|)}^{2}}$$

The *DRMSD* has already been shown to be a proper order parameter to study the folding process^23^. *DRMSD* = 0 corresponds uniquely to the target structure. The closer the global minimum is to *DRMSD* = 0, the smaller is the corresponding ensemble of structures. Thus, if the free energy landscape has a clear global minimum close to *DRMSD* = 0, we can identify at least one pattern that drives the system to fold into a unique target structure: the polymer is designable. Based on this qualitative definition we already clearly separate two groups: one with global minimum with position *DRMSD* < 0.6 and one with global minimum in the range $DRMSD\in [0.8,1.4]$.

However, in order to define more precisely a threshold we increase the temperature and push the system to unfold. Upon increasing the temperature the minimum significantly shifts towards higher *DRMSD* (as in Supp. Mat. Fig. 2), consistently with a temperature induced folding-unfolding transition. In some cases, our temperature resolution was high enough to observe two simultaneous minima. The configurations with *DRMSD* values corresponding to the position of the second minimum are the molten globule structures. Interestingly, for the same alphabet size the position of the molten globule minimum is conserved for different number of patches (see Supp. Mat. Fig. 2), and for 0 patches is the only global minimum observed at all temperatures (as in Supp. Mat. Fig. 2). Hence, the 0 patch chain was never designable. Thus, we choose as definition for the threshold between folded and not folded for each alphabet size (grey area in Fig. 4) the *DRMSD* range spanning 1*K*_*B*_*T* from the position of the global minimum of the free energy of the case with 0 patches. Systems with global minimum at any temperature below this area are labeled as designable, the others as non-designable.

We observed from the simulations that the position of the minimum corresponding to the molten globule does not change significantly with the number of patches while it varies with the alphabet size. The latter is because the bare heteropolymer without directional interactions, although it never folded, starts to feel the influence of the larger alphabets on its designability. Even when the system does not reach the folded state for the target structure that we identified via the SEEK, and hence the SDF trials fail, it might still be possible to find a handful of structures that are designable. However, since a heteropolymer with few and hard-to-find designable structures is not a good candidate for potential applications, we label it as not-designable.

In all Monte Carlo simulations we enhance the sampling with the Virtual Move Parallel Tempering algorithm^63^, performing each simulation at 16 different temperatures in the set [3, 2.5, 2.0, 1.6, 1.4, 1.2, 1.0, 0.9, 0.8, 0.75, 0.7, 0.65, 0.6, 0.55, 0.5, 0.4]. The SEEK, DESIGN and FOLDING steps are each composed by 10 independent simulations, run until we observe that the free energy landscape does not vary anymore and that all the 10 independent simulations give the same results, i.e. the free energy surfaces for each of the 10 independent simulations overlap within the statistical error. So each time we categorise a minimum in the FOLDING free energy landscape as folded or non-folded, the minimum is found always at the same position over 10 independent simulations.

For the radial distribution functions of proteins, the normalisation has been performed on the same ideal gas with an average density, to make the *g*(*r*) of proteins with different lengths comparable. All the *g*(*r*) have been calculated by neglecting the contribution of the beads (or amino acids) directly connected to each other along the chain, in order to ignore their trivial contribution to the first neighbour’s peak.

### Data Availability

The datasets published in the current study are available in the website of our research group, http://homepage.univie.ac.at/ivan.coluzza/Home_Page/News/Entries/2017/5/13_Supplementary_data_for_The_role_of_directional_interactions_in_the_designability_of_generalized_heteropolymers.html. While the codes and the files necessary to launch are available in the git-bucket repository, https://bitbucket.org/viennafolding/bionic-proteins.

## Electronic supplementary material

SUPPLEMENTARY MATERIAL
