# Author Correction: The role of directional interactions in the designability of generalized heteropolymers

**DOI:** 10.1038/s41598-018-22649-3

**Published:** 2018-03-12

**Authors:** Chiara Cardelli, Valentino Bianco, Lorenzo Rovigatti, Francesca Nerattini, Luca Tubiana, Christoph Dellago, Ivan Coluzza

**Affiliations:** 1 Faculty of Physics, University of Vienna, Boltzmanngasse 5, A-1090 Vienna, Austria; 2 Rudolf Peierls Centre for Theoretical Physics, University of Oxford, 1 Keble Road, Oxford, UK

Correction to: *Scientific Reports* 10.1038/s41598-017-04720-7, published online 10 July 2017

This Article contains typographical errors in the Acknowledgements section.

“We acknowledge support from the Austrian Science Fund (FWF) project P23846-N16,”

should read:

“We acknowledge support from the Austrian Science Fund (FWF) project P26253-N27”

