# Supplementary material for: The role of directional interactions in the designability of generalized heteropolymers
Source: Sci Rep. 2017 Jul 10;7:4986. doi: 10.1038/s41598-017-04720-7 (PMC5504045; doi:10.1038/s41598-017-04720-7)
Supplement: Supplementary file 1 — SUPPLEMENTARY MATERIAL [file 41598_2017_4720_MOESM1_ESM.pdf]

The role of directional interactions in the designability of generalized heteropolymers - SUPPLEMENTARY MATERIAL

Chiara Cardelli[1], Valentino Bianco[1], Lorenzo Rovigatti[1,2], Francesca Nerattini[1], Luca Tubiana[1], Christoph Dellago[1], and Ivan Coluzza[1*]

[1] *Faculty of Physics, University of Vienna, Boltzmanngasse 5, A-1090 Vienna, Austria*

[2] *Rudolf Peierls Centre for Theoretical Physics, University of Oxford, 1 Keble Road Oxford, UK*

[*] ivan.coluzza@univie.ac.at

Supplementary theory

A guiding theory for heteropolymer designability is given by the Random Energy Model (REM) [1–3]. A clear review can be found in the seminal works of Pande *et al.* [3], where it is shown how the designability of a heteropolymer increases with the total number of possible bonds for each bead (valence) and decreases with the conformational entropy per bead. Hence, it is reasonable to assume that directionality (the patches) combined with isotropic interactions would increase designability, because the valence (i.e. the total number of possible bonds) remains constant, while the conformational entropy per bead decreases. In fact, the introduction of the patches decreases the entropy by favouring the system to populate more specific structures with the patches along particular directions.

On the other hand, if the number of patches increases to much, the interactions become again close to isotropic and the designability decreases again. Hence, a model is needed that explicitly brings about the designability from a basic heteropolymer model by controlling the alphabet size and the conformational entropy per particle.

Symmetry of patches on the surface

Considering n as the number of patches on the surface, for freely jointed chains (FJC) with $n = 3$ the anchoring points are places perpendicular to the patch, while for $n = 4$ they are placed on a tetrahedron completed by the anchoring points. The freely rotating chains (FRC) are fixed on the vertices of a platonic solid or in the most symmetric way: equispaced on the equator and placed on the vertices of a equilateral triangle, of a tetrahedron and of a octahedron for the $n = 3$ $n = 4$ $n = 6$ cases, respectively. For $n = 10$ there is no platonic solid, so the patches are placed on the surface in the most symmetric way by using the following numerical procedure:

1. n patches are randomly placed on a sphere, their positions given by the set of vectors $\{\vec{r_1}, \ldots, \vec{r_n}\}$
2. we assign a fictitious energy to the system, defined as $U = \frac{1}{2}\sum_{i\neq j} |\vec{r_i} - \vec{r_j}|^{-1}$
3. we minimise U by attempting to move a randomly chosen patch, accepting the move if the total energy of the system consequently decreases. Formally, this can be regarded as a Monte Carlo (MC) simulation performed at temperature zero.
4. We iterate this procedure until convergence of U.

For completeness, we note that the above method produces a patch distribution which is independent of the definition of distance between two patches, being it the Euclidean distance or a spherical distance, for all the values of n considered here. In addition, the patch distribution makes sure that two particles cannot be involved in more than one bond.

Supplementary results figures

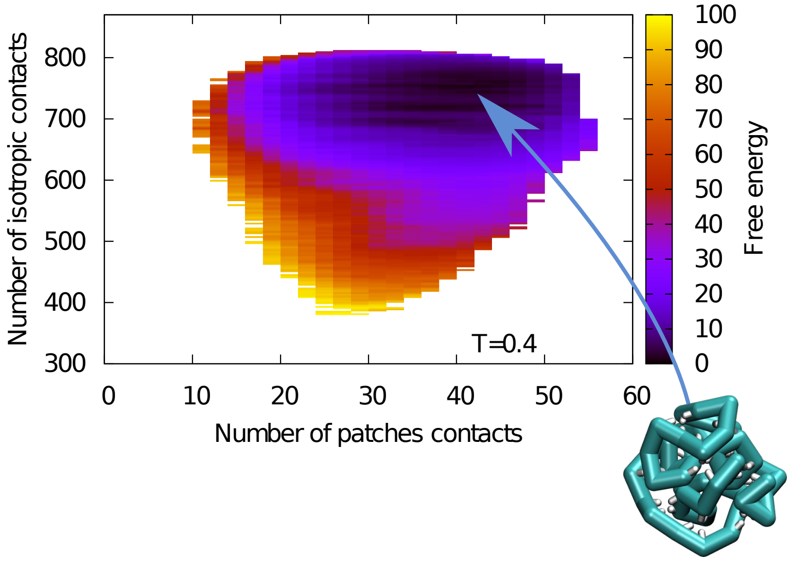

FIG. S.1. Free energy landscape sampled by SEEK for one patch and alphabet 3 in the freely rotating chain model. The free energy is in function of the total number of contacts between the spheres (distance below 6 R_{bead}) and the total number of contacts between the patches (distance below 1.25 R_{bead} and angles θ_1 and $\theta_2 > 0.8\ \pi$). The target structure is chosen in the global minimum of this landscape. Following the above definition of patches contacts, in the target structure the 80% of the patches are maximally oriented between each others. However, we observe by looking closer at the structure, that all the patches are interacting. Nevertheless, the close packing peak in the radial distribution function dominates on the directional interaction peak. Hence, the close packing is not suppressed even when all the directional interactions are fulfilled. Thus, increasing the relative strength of the directional interactions will not make the first peak disappear.

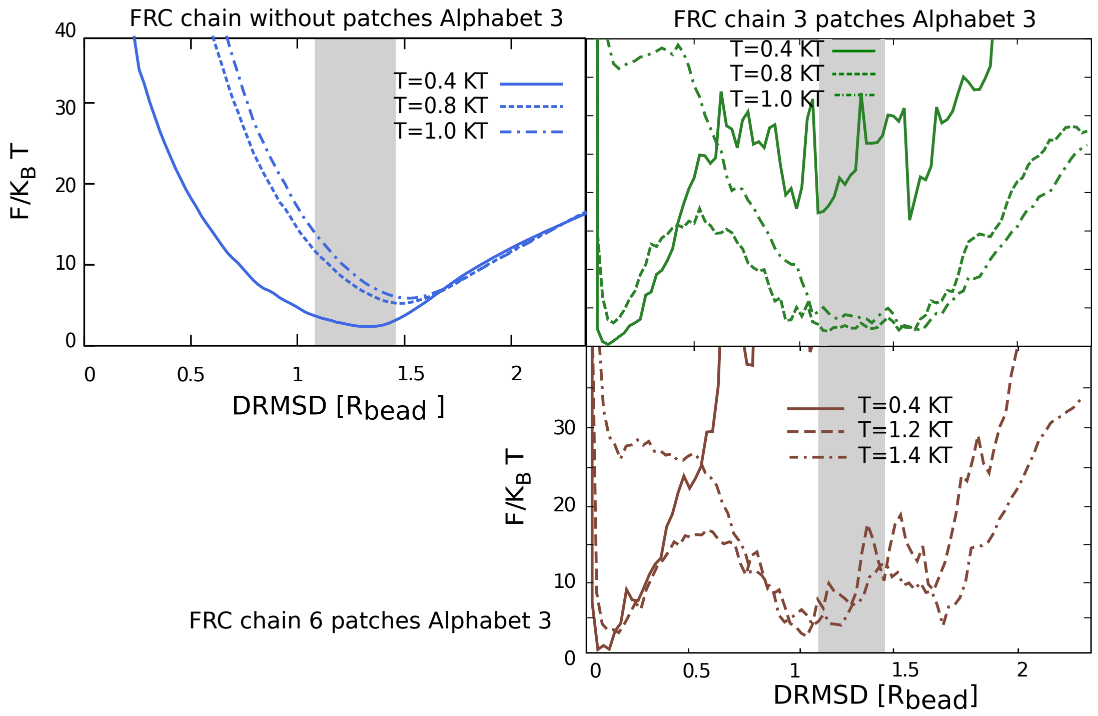

FIG. S.2. FOLDING free energy landscape as a function of the distance root mean square displacement ($DRMSD$) for an example of a non-designable system (left) and two designable ones (right). In the cases on the right, our temperature resolution was high enough to observe two simultaneous minima. The configurations with $DRMSD$ values corresponding to the position of the second minimum are the molten globule structures. Interestingly, for the same alphabet size the position of the molten globule minimum is conserved for different number of patches, and for 0 patches is the only global minimum observed at all temperatures. Hence, the 0 patch chain is never designable.

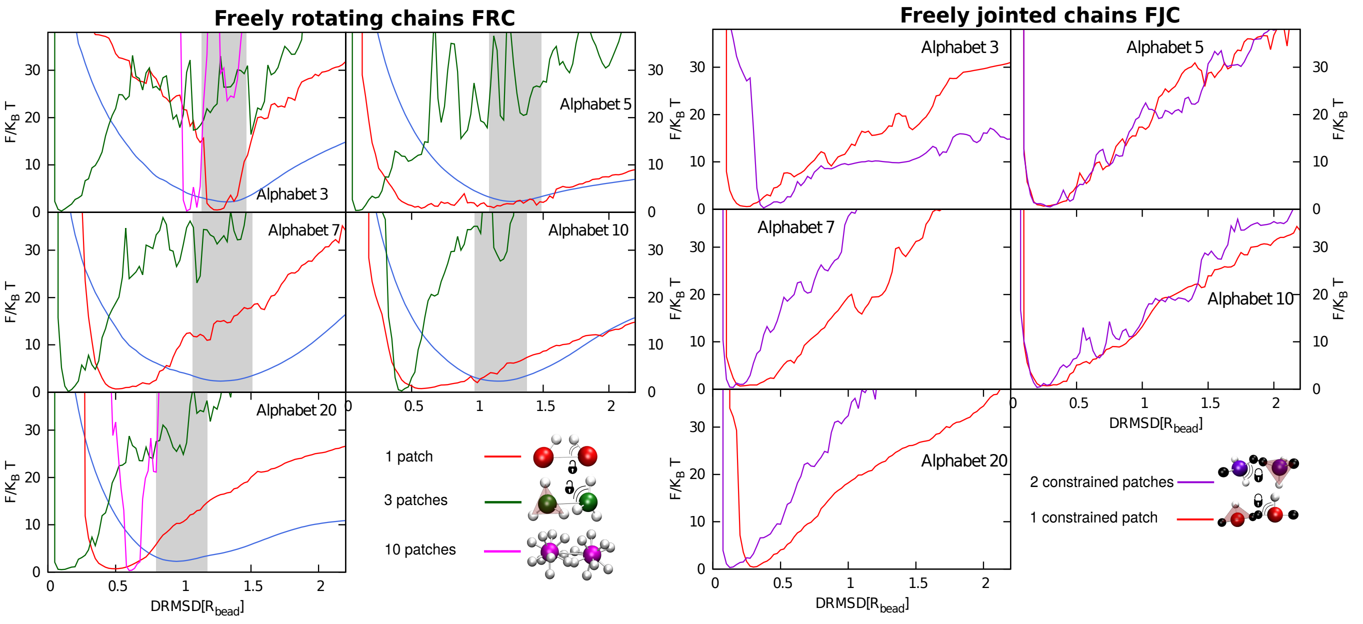

FIG. S.3. FOLDING free energy landscapes. The free energy is plotted as a function of the distance root mean square displacement ($DRMSD$) for freely rotating chain (left) and freely jointed chain (right), for different patches numbers and alphabet sizes at temperature 0.4.

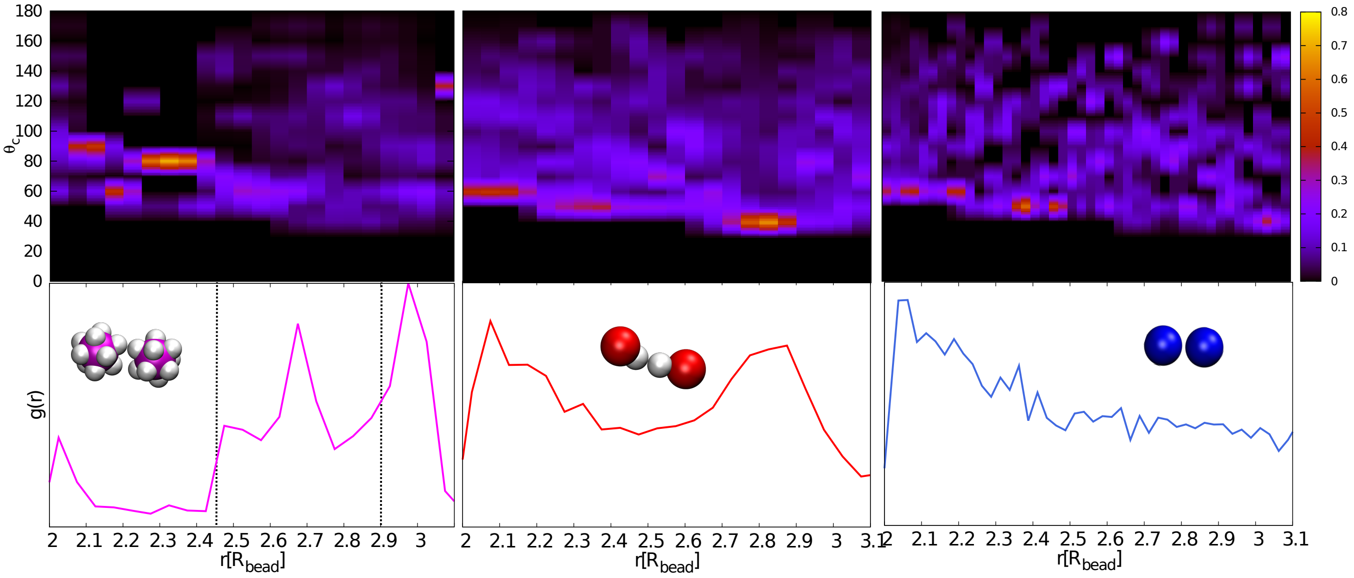

FIG. S.4. Probability that one bead forms an angle θ_c with two other beads located at distance r from the central one. The probability is averaged on the 40 most probable conformations and we exclude the angles formed by three consecutive beads along the chain. a) without patches. b) 10 patches.

For the polymer without patches we observe in Figure S.4 that the prevalent angle is $60°$ corresponding to a random packing. For 10 patches we observe that the high number of patches decreases the random packing at distances close to $2R_{bead}$. In fact, at small distances $(2.00 - 2.45R_{bead})$ there is a component of directionality that cannot be attributed to the patch-patch bond, but it is instead due to the inter-penetration of the corona formed by the 10 patches, which can occur only with discrete angles. While at intermediate distances $(2.45 - 2.90R_{bead})$ be observe that the peak at $60°$ reappears. In fact, this correspond to the new random packing, which is shifted due to the hindrance of the corona formed by the many patches. In the last part we observe only at distances $3R_{bead}$ the reappearing of some directionality due to the patch-patch bond.

[1] Gutin, A. M.; Shakhnovich, E. *The Journal of Chemical Physics* **1993**, *98*, 8174–8177.
[2] Shakhnovich, E. I.; Gutin, A. M. *Proceedings of the National Academy of Sciences of the United States of America* **1993**, *90*, 7195–9.
[3] Pande, V. S.; Grosberg, A. Y.; Tanaka, T. *Reviews of Modern Physics* **2000**, *72*, 259–314.

Interaction matrix for alphabet size 3

	1	2	3
1	0.405427	0.399805	-0.408428
2		0.083519	-0.185762
3			-0.294699

	1	2	3	4	5
1	0.073272	-0.056012	-0.176851	0.055725	0.072086
2		0.115497	-0.358047	-0.606938	-0.02605
3			0.327363	0.441482	-0.221434
4				-0.003289	-0.046151
5					0.417604

Interaction matrix for alphabet sizes 7

	1	2	3	4	5	6
1	-0.488145	-0.099172	-0.665188	-0.513159	-0.140222	0.027036
2		-0.411612	0.731346	0.494311	0.067639	0.39224
3			0.339552	-0.428898	-0.463306	-0.061358
4				0.356201	-0.377746	0.60377
5					-0.035632	0.013969
6						0.581666
7						

Interaction matrix for alphabet sizes 7

7
-0.023432
0.06542
0.325872
-0.715795
0.282897
0.402024
-0.279583

Interaction matrix for alphabet size 10

	1	2	3	4	5	6
1	0.036338	-0.109579	0.450045	0.211862	-0.144162	-0.156263
2		-0.014035	0.001334	0.219005	0.672716	0.174732
3			-0.422396	0.2583	-0.064613	0.015065
4				0.341895	-0.130257	-0.503189
5					0.320228	-0.077332
6						-0.151698
7						
8						
9						
10						

Interaction matrix for alphabet size 10

7	8	9	10
-0.06087	0.335178	0.383069	0.398842
0.117673	-0.097265	-0.038571	0.267376
0.767522	-0.068141	-0.099905	-0.223041
0.175065	-0.009685	-0.07783	0.319185
0.072921	-1.035992	-0.465169	-0.18564
-0.016638	-0.029539	-0.19378	-0.247656
-0.049106	0.12019	0.448171	-0.742203
	0.023326	-0.221476	-0.065685
		-0.173327	-0.215073
			-0.022223

Interaction matrix for alphabet size 20

	1	2	3	4	5	6
1	0.081553	0.426631	0.568848	-0.502789	-0.12822	-0.435738
2		0.353419	-0.097927	0.389741	0.243083	-0.336347
3			-0.056157	-0.179468	0.111268	-0.135234
4				0.701963	-0.041826	0.388943
5					-0.073145	-0.186174
6						0.553226
7						
8						
9						
10						
11						
12						
13						
14						
15						
16						
17						
18						
19						
20						

Interaction matrix for alphabet size 20

7	8	9	10	11	12	13
-0.348879	0.128948	0.045576	-0.28945	0.048781	-0.354339	0.273338
0.166491	-0.072994	0.017063	-0.324963	0.169753	0.027633	-0.870442
-0.029389	-0.079183	-0.235617	0.197951	-0.698542	0.131127	0.061888
0.534033	0.275594	-0.097178	0.125153	0.246242	-0.430059	-0.530827
0.016316	0.411457	-0.402362	0.49164	-0.031645	0.034439	-0.129541
0.485911	0.142607	0.089138	-0.26214	-0.30248	0.237108	-0.528338
-0.078432	0.291858	0.03107	-0.038642	0.480908	-0.198523	0.357243
	0.392551	-0.059625	0.200538	-0.468885	-0.05802	-0.284558
		-0.117411	0.081203	-0.173082	-0.186838	-0.626013
			0.084789	0.045141	-0.120325	-0.037095
				-0.161365	-0.352376	0.066567
					-0.000727	0.179239
						0.355301

Interaction matrix for alphabet size 20

14	15	16	17	18	19	20
0.900379	-0.305614	0.11782	0.141584	0.194476	-0.021256	-0.111908
0.125427	0.342717	0.099973	-0.244877	-0.357507	0.319781	-0.236908
-0.619739	0.36774	-0.161619	-0.18599	-0.678679	-0.142067	-0.003966
-0.093322	-0.433012	0.29192	0.010121	-0.627608	0.323705	0.174866
0.026183	-0.129373	-0.034842	-0.494044	0.381081	0.323671	0.046249
0.500438	-0.027833	0.357806	-0.29547	-0.524512	-0.240258	-0.504283
0.1966	-0.153644	0.765287	0.442432	-0.286014	0.227902	0.466241
-0.096826	0.311937	0.000396	0.147358	0.395159	0.372012	0.362592
0.220451	0.073642	0.060668	0.383668	-0.593914	-0.303292	0.074573
0.01467	0.217459	-0.630983	-0.432735	-0.09005	-0.205353	0.056339
0.287437	-0.296898	-0.029977	-0.803003	0.255198	-0.546963	-0.122039
-0.169458	0.109036	0.237348	0.381594	0.008841	-0.102676	-0.091471
-0.186524	0.253168	-0.127866	0.090694	0.031939	-0.376384	-0.007257
-0.152785	-0.266961	-0.094049	-0.276912	-0.452899	0.025443	-0.4211
	-0.275975	0.667552	0.354217	-0.136487	0.086701	0.174718
		0.003189	0.086487	0.135358	-0.01824	0.346642
			-0.223676	-0.102268	0.362454	0.215395
				-0.067085	0.173723	0.679878
					0.33719	-0.109486
						0.084862
